# Associations between perceived value of exclusive breastfeeding among pregnant women in the United States and exclusive breastfeeding to three and six months postpartum: a prospective study

**DOI:** 10.1186/s13006-016-0065-x

**Published:** 2016-04-12

**Authors:** Uche H. Nnebe-Agumadu, Elizabeth F. Racine, Sarah B. Laditka, Maren J. Coffman

**Affiliations:** Affiliated with the Department of Public Health Sciences, University of North Carolina at Charlotte, 9201 University City Blvd, Charlotte, NC 28223 USA; Department of Public Health Sciences, University of North Carolina at Charlotte, 9201 University City Blvd, Charlotte, NC 28223 USA; School of Nursing, College of Health and Human Services, University of North Carolina at Charlotte, 9201 University City Blvd, Charlotte, NC 28223 USA

**Keywords:** Exclusive breastfeeding, Breastfeeding duration, Value breastfeeding, Prenatal and postnatal breastfeeding education

## Abstract

**Background:**

Successful breastfeeding often starts with prenatally established intention. Yet, few mothers with the intention to exclusively breastfeed achieve their intended breastfeeding duration goal. This study examined the degree to which having a strong value of exclusive breastfeeding is associated with exclusive breastfeeding duration for at least 3 and 6 months postpartum among women who reported prenatal intention to exclusively breastfeed.

**Methods:**

Data were from the Infant Feeding Practices Study II, a longitudinal US national survey that followed maternal-infant dyads from pregnancy to 1 year postpartum. Bivariate and multivariate regression examined the degree to which strong maternal value of exclusive breastfeeding predicted exclusive breastfeeding duration.

**Results:**

Of the 1799 women who prenatally intended to exclusively breastfeed within the first few weeks postpartum, 34 and 9 % exclusively breastfed for at least 3 months and 6 months, respectively. Thirty-six percent of women reported strongly valuing exclusive breastfeeding out of which 46 % exclusively breastfeed to three months. In adjusted results, women who reported that they strongly value exclusive breastfeeding had more than twice the odds of exclusive breastfeeding for at least 3 months (Adjusted Odds Ratio [AOR] 2.29; 95 % confidence interval [CI] 1.84, 2.85) and for 6 months (AOR 2.49; 95 % CI 1.76, 3.53) compared to those who did not strongly value exclusive breastfeeding.

**Conclusion:**

Valuing the benefits of exclusive breastfeeding during pregnancy is a strong independent predictor of actual exclusive breastfeeding duration. A minority of pregnant women strongly value exclusive breastfeeding and are able to exclusively breastfeed to 3 months even among women with established prenatal intention to exclusively breastfeed. Prenatal maternal education and environmental lactation support that extends into the postnatal period can promote longer duration of exclusive breastfeeding.

## Background

Breastfeeding promotion is an important area for public health intervention because of low rates of exclusive breastfeeding duration past a few weeks post-partum [1]. In the United States, the American Academy of Pediatrics recommends exclusive breastfeeding from birth for 6 months followed by continued breastfeeding as complementary foods are introduced, with continuation of breastfeeding for 1 year or longer as desired by mother and infant [2]. A relatively small percentage of women breastfeed exclusively as recommended and the proportion of infants who are exclusively breastfed for 6 months after birth has increased at a much slower rate compared to that of infants who receive mixed feeding [3, 4]. Reasons for this slow improvement among mothers in the United States include: (a) breastfeeding has not received sufficient national attention as a public health issue [5]; (b) inadequate attention to the importance of the duration of exclusive breastfeeding [3, 6]; and (c) inadequate research addressing exclusive breastfeeding in the United States [3, 7].

Among infants born in 2011 in the United States, only 41 and 19 % were breastfed exclusively for 3 months and 6 months respectively [8]. Exclusive breastfeeding was measured from birth but included babies who received infant formula the first 2 days of life only. Low exclusive breastfeeding rates have been reported in many countries throughout the world. Currently, about 38 % of children worldwide who are under 6 months of age are exclusively breastfed from birth; this percentage has not increased notably in the past two decades [9]. The highest exclusive breastfeeding rates are reported in eastern and southern regions in Africa (51 %) [9].

Intention to breastfeed is the most critical determinant of breastfeeding, particularly exclusive breastfeeding [3, 10]. Nevertheless, when mothers intend to breastfeed, few are able to achieve their intended breastfeeding duration [11]. Reported barriers include maternal work [12–14], age [11, 15], available emotional support, especially support of the baby’s father [16], planned pregnancy and delivery [13], race [4, 17, 18] and maternal education [15, 18, 19]. Studies also reported individual constraints, especially the perception of having insufficient milk for the baby [11, 20] and the inconvenience of exclusive breastfeeding especially for mothers returning to work [11, 20, 21]. Some mothers report feeling embarrassed by breastfeeding in public places as well as being concerned about having their baby get too attached to them [22, 23].

Addressing barriers through maternal education may help to improve the percentage of women who are able to exclusively breastfeed for a longer duration. The United States Breastfeeding Committee [6] reported misperceptions and fears of exclusive breastfeeding as barriers to exclusive breastfeeding. The Committee recommended increasing awareness and attention of the importance of exclusive breastfeeding to increase exclusive breastfeeding rates. Research has shown that mothers with more education are more likely than those with less education to exclusively breastfeed for longer durations [24, 25]. In addition, mothers who are educated about the benefits of exclusive breastfeeding are more likely to value exclusive breastfeeding and may be more determined to breastfeed for longer durations [15, 26].

Our study extends previous research by examining and quantifying maternal value of exclusive breastfeeding as a determinant of exclusive breastfeeding for at least 3 months and 6 months. Little research has examined the role of psychosocial factors of exclusive breastfeeding as they relate to maternal knowledge of exclusive breastfeeding benefits. Maternal psychological factors are more strongly associated with breastfeeding duration than sociodemographic factors [19]. A better understanding of maternal factors associated with exclusive breastfeeding can help to develop more effective ways to increase exclusive breastfeeding duration.

We examined mothers’ intention to exclusively breastfeed to determine whether strongly valuing exclusive breastfeeding can predict breastfeeding exclusively to 3 and to 6 months, using the attitude (or value) of mothers toward breastfeeding as a main predictor. An established intention to breastfeed is a well-established determinant of breastfeeding duration. Thus, our analysis was limited to pregnant women who reported that they intended to exclusively breastfeed in the first few weeks. Despite an established intention, a relatively small percentage of mothers attain their desired or recommended duration of exclusive breastfeeding. Identifying and overcoming these barriers can enhance breastfeeding practices. We hypothesized that among mothers who intend to exclusively breastfeed, those who strongly value exclusive breastfeeding will be more likely to breastfeed exclusively for at least 3 months and 6 months compared to mothers who value exclusive breastfeeding less. We examined exclusive breastfeeding duration to both 3 months and 6 months as we expected that fewer mothers would exclusively breastfeed to 6 months, which would limit the power of the analysis. Focusing on exclusive breastfeeding, this study highlights the importance of the duration of exclusive breastfeeding and adds to our understanding of maternal personal factors that promote exclusive breastfeeding.

## Methods

This was a secondary analysis of the United States Infant Feeding Practices Study II (IFPS II), a longitudinal survey of mothers of healthy singleton pregnancies conducted by the Food and Drug Administration (FDA) in collaboration with the Centers for Disease Control and Prevention (CDC) [27]. The IFPS II is a convenience sample of 4,902 mothers in their third trimester recruited through a nationally distributed consumer-opinion panel of over 500,000 households from May 2005 through June 2007. Information was collected from mothers using a series of questionnaires administered from the seventh month of pregnancy through the infant’s first year of life. Each participant was mailed one prenatal and ten postnatal questionnaires. A neonatal questionnaire was sent when the infant was 3 weeks old; nine questionnaires about infant feeding, health care, and related topics were sent monthly when infants were ages 2 to 7 months and then approximately every 7 weeks until the infant was 12 months old. The mothers also received a demographic questionnaire. Mother-infant dyads qualified to be included in the sample if mothers were at least 18 years old, and infants were born after 35 weeks of gestation or more and weighed at least five pounds at birth, were singleton and healthy. Infant health was measured by: whether an infant stayed in an intensive care unit for more than 3 days or whether an infant had special needs or medical problems based on a pediatrician evaluation [28]. The survey excluded mothers and infants who had serious long-term health problems that would interfere with infant feeding, such as Down’s syndrome and cleft palate.

We analyzed data from prenatal and postnatal questionnaires. Each questionnaire had an average of 50 questions and took from 10 to 30 min to complete depending on the number of questions answered [27]. Prenatal questionnaires were sent to 14,618 mothers; 4,902 mothers completed and returned the surveys. A response rate was not reported because the returned questionnaires were from qualified mother-infant dyads only. It was not possible to determine the correct estimate of non-respondents who did not qualify for the study because mothers were disqualified for one or a combination of reasons; data showing how these criteria overlap are not available [27]. After the baby was born, 3,033 mothers were sent questionnaires, with a response rate of 63 to 83 % [27].

Our analysis was limited to pregnant mothers who reported that they intended to exclusively breastfeed in the first few weeks. This information was obtained from the following question in the prenatal questionnaire: “What method do you plan to use to feed your new baby in the first few weeks?” Mothers who answered “breastfeeding only” were in our analytic sample (*n* = 2781). Other response options were: “formula feed only”, “both breast and formula feed”, and “don’t know yet”.

The exposure variable was “value of exclusive breastfeeding”. This variable was measured from the prenatal questionnaire using the question: “How strongly do you agree or disagree with the following statements? “Babies should be exclusively breastfed for the first 6 months”. Answer options were: “Strongly disagree”, “somewhat disagree”, “neither agree nor disagree”, “somewhat agree”, “strongly agree”. Mothers who responded “strongly agree” were considered to “strongly value exclusive breastfeeding”. We interpret this as a valid measure of maternal attitude. Other categories were collapsed to form “does not strongly value exclusive breastfeeding”.

“Duration of exclusive breastfeeding” was the outcome variable. Duration was measured from birth and at each postnatal follow-up questionnaire. In each survey mothers were asked the feeding method used in the past 7 days. The responses were used by researchers at the FDA and CDC to calculate duration of exclusive breastfeeding. We used this information to determine whether the mother breastfed exclusively for at least 13 weeks (3 months) or for 26 weeks (6 months). Exclusive breastfeeding was defined as providing the infant only breast milk and restricting any additional food or drink including water with the exception of medicines, vitamins and mineral supplements.

Information about covariates was obtained from the demographic, prenatal and neonatal questionnaires. We included control variables consistent with relevant studies: maternal age (18–24, 25–35, ≥ 35 years) [6, 11], marital status (married and unmarried) [6, 29], maternal education (high school graduate or less, some college education, and college graduate or higher) [19]. Income was (<185 % of Federal Poverty Level [FPL], 185–349 % of FPL and ≥ 350 % of FPL) based on federal poverty guidelines for 2006 published by the United States Census Bureau. Other controls were: parity (primipara and multipara) [19, 29], maternal prenatal employment (yes/no) [12, 13], prenatal smoking (yes/no) [30, 31], delivery mode (vaginal, planned cesarean section, emergency cesarean section) [32, 33], and the father’s support for exclusive breastfeeding (yes/no) [13, 16].

Race/ethnicity [4, 17] was categorized as white and non-white. Black, Hispanic and “others” representing Asians, Pacific Islanders and mothers in other racial minorities were grouped together as non-white because of their few numbers (Black = 4 %, Hispanic = 6 %, other racial minorities = 5 %). Total maternity leave taken was based on responses to two questions: “Do you plan to work for pay during babies first year?” and if the response was yes, mothers were asked; “how many weeks after birth do you plan to return to work?” We created two categories: mothers who planned to return to work in less than 6 weeks and mothers who planned to return to work 6 weeks postpartum. Mothers who did not intend to work for pay in the year following delivery formed a third category. Mothers who intended working for pay but did not indicate how many weeks after birth they intend to return to work and those with missing data were grouped together (*n* = 307, 16 %). We calculated body mass index (BMI, kg/m^2^) using information from prepregnancy weight and height. [34, 35]. BMI was then dichotomized into obese (BMI ≥ 30) and non-obese (BMI < 30).

We used descriptive statistics to examine demographic and other characteristics of mothers who reported that they intended to exclusively breastfeed. Differences in characteristics between mothers who exclusively breastfed for 3 months and 6 months and those who did not were examined using the chi-square tests. Unadjusted associations between maternal value of exclusive breastfeeding and exclusive breastfeeding duration for at least 3 months and 6 months were examined using logistic regression. The covariates were also tested against the outcome variables for significant associations. Separate multivariate logistic regression models were used to evaluate the impact of maternal value of exclusive breastfeeding on exclusive breastfeeding duration for 3 months and for 6 months controlling for covariates that were significant at *p* < 0.05 in the bivariate analyses. Statistical significance was determined at the *p* < 0.05 level for all analyses.

The Institutional Review Board (IRB) at the University of North Carolina at Charlotte, determined that this research, which used de-identified secondary data from a publicly available dataset, did not require IRB review.

## Results

Of the 4902 pregnant mothers in the IFPS II, 2781 (58.6 %) reported a prenatal intention to exclusively breastfeed in the first few weeks postpartum. In the postnatal period, 976 (28.7 %) were lost to follow up, reducing the sample size to 1895. Six mothers with missing data on the exposure variable further reduced the sample to 1799. Of the 1799 mothers who intended to exclusively breastfeed in the first few weeks, 157 (9 %) breastfed exclusively for 6 months; 618 (34 %) breastfed exclusively for at least 3 months. Most mothers were white (87 %), did not smoke (94 %), were married (82 %), were multiparous (67 %) and had more than a high school education (84 %) (Table 1). Other than income, all of the covariates were significantly different for mothers who breastfed for 3 months than those who did not. For example, a higher proportion of mothers who exclusively breastfed for at least 3 months were married, older, non-smokers, more educated, less likely to have a cesarean delivery, and strongly valued exclusive breastfeeding compared to mothers who did not exclusively breastfeed for at least 3 months (all *p* = 0.0001). Only 36 % of the mothers reported that they strongly valued exclusive breastfeeding. Of these, 46 % (298/647) exclusively breastfed for at least 3 months and 14 % (90/647) exclusively breastfed for at least 6 months (Table 1). Among mothers without the intention to exclusively breastfeed (1099), only 6 % reported strong value of exclusive breastfeeding; 3 and 0.6 % exclusively breastfed to 3 months and 6 months respectively (results not shown in tables).

**Table 1:** Sample characteristics of mothers who intended to exclusively breastfeed, by actual breastfeeding duration^a^

Characteristics	Full sample	Exclusive breastfeeding					
		≥ 3mos	≤ 3 mos	*p*-values^b^	≥ 6mos	≤ 6 mos	*p*-values^b^
	*N* = 1799 (%)	*n* = 618 (%)	*n* = 1181 (%)		*n* = 157 (%)	*n* =1642 (%)	
Strongly value EBF							
Yes	647 (36.0)	298 (48.2)	349 (29.6)	<.0001	90 (57.3)	557 (33.9)	<.0001
No	1152 (64.0)	320 (51.8)	832 (70.5)		67 (42.7)	1085 (66.1)	
Married							
Yes	1410 (82.7)	564 (91.7)	864 (78.0)	<.0001	137 (93.2)	285 (18.3)	.0017
No	295 (17.3)	51 (8.3)	244 (22.0)		10 (6.8)	1273 (81.7)	
Prenatal smoking							
Non- smokers	1691 (94.0)	603 (97.6)	1088 (92.1)	<.0001	153 (97.4)	1538 (93.7)	.0564
Current smokers	108 (6.0)	15 (2.4)	93 (7.9)		04 (2.6)	104 (06.3)	
Parity							
Primipara	582 (33.0)	133 (21.6)	449 (39.0)	<.0001	35 (22.6)	547 (34)	.0118
Multipara	1181 (67.0)	479 (78.4)	702 (61.0)		120 (77.4)	1061 (66)	
Prenatal employment							
No	719 (45.6)	285 (50.7)	434 (42.8)	<.0001	80 (58)	639 (44.4)	.0092
Yes	858 (54.4)	277 (49.3)	581 (57.2)		58 (42)	800 (55.6)	
Race/Ethnicity							
White	1528 (87.2)	550 (90.9)	978 (85.2)	.0021	138 (90.8)	1390 (86.8)	.3304
Non- White	225 (12.8)	55 (9.1)	170 (14.8)		14 (9.2)	211 (13.2)	
Income status							
< 185 % of FPL^c^	656 (36.5)	234 (37.9)	422 (35.7)	0.36	70 (44.6)	586 (35.7)	.0429
185 %–349 % of FPL	690 (38.4)	223 (36.1)	467 (39.6)		47 (29.9)	643 (39.2)	
> 350 % of FPL	453 (25.2)	161 (26.0)	292 (24.7)		40 (25.5)	413 (25.1)	
Maternal age							
18–24 years	477 (26.5)	107 (17.4)	370 (31.3)	<.0001	32 (20.5)	445 (27.1)	.2022
25–34 years	1037 (57.7)	400 (64.8)	637 (53.9)		98 (62.8)	939 (57.2)	
≥ 35 years	284 (15.8)	110 (17.8)	174 (14.8)		26 (16.7)	258 (15.7)	
Maternal education							
≤ High school	264 (15.6)	70 (11.7)	194 (17.6)	<.0001	15 (10.3)	249 (16.0)	.2139
Some college	679 (40.0)	210 (35.3)	469 (42.6)		61 (42.1)	618 (39.8)	
≥ College graduate	754 (44.4)	316 (53.0)	438 (39.8)		69 (47.6)	685 (44.2)	
Plans to return to work							
≤ 6 weeks	176 (11.8)	51 (9.7)	125 (12.9)	<.0001	05 (4.6)	171 (12.4)	<.0001
> 6 weeks	712 (47.7)	209 (39.9)	503 (52.0)		28 (25.7)	684 (49.4)	
Did not plan to work	604 (40.5)	264 (50.4)	340 (35.1)		76 (69.7)	528 (38.2)	
	Full Sample	≥ 3mos	≤ 3 mos	*p*-values^b^	≥ 6mos	≤ 6 mos	*p*-values^b^
Delivery mode							
Vaginal	1326 (73.71)	489 (79.13)	837 (70.87)	<.0001	127 (80.9)	1199 (73.0)	.02
Planned C-section	254 (14.12)	83 (13.43)	171 (14.48)		22 (14.0)	232 (14.1)	
Emergency C-section	219 (12.17)	46 (7.44)	173 (14.65)		8 (5.1)	211 (12.9)	
Baby’s father support							
No	412 (22.9)	89 (14.4)	323 (27.3)	<.0001	18 (11.5)	394 (24)	.0004
Yes	1387 (77.1)	529 (85.6)	858 (72.7)		139 (88.5)	1248 (76)	
Maternal obesity							
Not obese	1372 (77.2)	497 (81.3)	875 (75.0)	0.0026	129 (83.2)	1243 (76.6)	.0616
Obese	405 (22.8)	114 (18.7)	291 (25.0)		26 (16.8)	379 (23.4)	

^a^Data source: Infant Feeding Practices Survey II, 2005–2007

^b^Chi-square was used to test for differences in characteristics between women who exclusively breastfed for at least 3 months and women who exclusively breastfed less than 3 months

^c^FPL = Federal Poverty Level: a measure of income level for determining financial eligibility for some welfare programs and benefits

In the unadjusted logistic regression analysis, the odds of exclusively breastfeeding for at least 3 months (Odds Ratio [OR] 2.22; 95 % confidence interval [CI]; 1.82, 2.72) and 6 months (OR 2.62; 95 % CI 1.88, 3.60) were significantly higher among mothers who strongly valued exclusive breastfeeding (Table 2). In the multivariate analysis, mothers who strongly valued exclusive breastfeeding had over 2 times the odds of exclusively breastfeeding for at least 3 months (Adjusted Odds Ratio [AOR] 2.29; 95 % CI 1.84, 2.85) and 6 months (AOR 2.49; 95 % CI 1.76, 3.53) compared to mothers who did not strongly value exclusive breastfeeding (Table 3).

**Table 2:** Unadjusted associations between exclusive breastfeeding duration and prenatal value of exclusive breastfeeding and other maternal characteristics among women who intended to exclusively breastfeed^a^

	3 mos EBF^b^ (*n* = 618)		6 mos EBF^b^ (*n* = 157)	
Characteristics	Odds ratio	95 % CI^c^	Odds ratio	95 % CI^c^
Strongly value EBF^b^				
No	1.00	Reference	1.00	Reference
Yes	2.22	1.82, 2.72	2.62	1.88, 3.60
Age				
18–24	0.46	0.36, 0.59	0.69	0.46, 1.04
25–35	1.00	Reference	1.00	Reference
≥ 35	1.01	0.77, 1.32	0.97	0.61, 1.52
Race/Ethnicity				
White	1.00	Reference	1.00	Reference
Non-Whites	0.58	0.42, 0.79	0.67	0.38, 1.18
Marital status				
Not married	1.00	Reference	1.00	Reference
Married	3.02	2.20, 4.17	3.07	1.59, 5.90
Maternal Education				
≤ High School	1.00	Reference	1.00	Reference
Some college	1.24	0.90, 1.71	1.64	0.91, 2.93
≤ College graduate	1.99	1.47, 2.72	1.67	0.94, 2.98
Prenatal employment				
Not employed	1.00	Reference	1.00	Reference
Employed	0.73	0.59, 0.89	0.58	0.41, 0.83
Poverty				
< 185 % of FPL^d^	1.00	Reference	1.00	Reference
185 %–349 % of FPL^d^	0.86	0.69, 1.08	0.61	0.42, 0.90
> 350 % of FPL^d^	0.99	0.77, 1.28	0.81	0.54, 1.22
Parity				
Multipara	1.00	Reference	1.00	Reference
Primipara	0.43	0.35, 0.54	0.57	0.38, 0.84
Prenatal smoking				
Current smokers	1.00	Reference	1.00	Reference
Non smokers	3.44	1.97, 5.98	2.59	0.94, 7.10
Baby’s father support				
No	1.00	Reference	1.00	Reference
Yes	2.24	1.73, 2.90	2.44	1.47, 4.04
Maternal obesity				
Non-obese	1.00	Reference	1.00	Reference
Obese	0.69	0.54, 0.88	0.66	0.43, 1.02
Delivery mode				
Vaginal delivery	1.00	Reference	1.00	Reference
Planned C-section	0.83	0.63, 1.11	0.90	0.56, 1.44
Emergency C-section	0.46	0.32, 0.64	0.36	0.17, 0.74
Plans to return to work				
≤ 6 weeks	1.00	Reference	1.00	Reference
> 6 weeks	1.02	0.71,1.47	1.40	0.53, 3.68
No plan to work	1.90	1.32, 2.74	4.90	1.96, 12.37

^a^Data source: Infant Feeding Practices Study II, 2005–2007

^b^EBF = Exclusive breastfeeding

^c^CI = confidence level

^d^FPL = Federal Poverty Level: a measure of income level for determining financial eligibility for some welfare programs and benefits

**Table 3:** Adjusted models of exclusive breastfeeding for 3 months and 6 months and prenatal value of exclusive breastfeeding^a^

	3 mos EBF^b^ (*n* = 618)		6 mos EBF^b^ (*n* = 157)	
Characteristics	AOR^c^	95 % CI^d^	AOR^c^	95 % CI^d^
Value of EBF				
No	1.00	Reference	1.00	Reference
Yes	2.29	1.84, 2.85	2.43	1.71, 3.44
Age				
18–24	0.68	0.51, 0.91	____	____
25–35	1.00	Reference	____	____
> 35	0.98	0.73, 1.32	____	____
Race/Ethnicity				
White	1.00	Reference	____	____
Non-Whites	0.61	0.43, 0.87	____	____
Marital status				
Not married	1.00	Reference	1.00	Reference
Married	1.62	1.13, 2.33	2.96	1.46, 5.99
Maternal education				
High School/ less	1.00	Reference	-----	------
Some college education	1.08	0.76, 1.53	-----	------
College graduate or more	1.55	1.08, 2.21	-----	------
Employment				
Not employed	1.00	Reference	1.00	Reference
Employed	1.01	0.78, 1.30	1.06	0.69, 1.62
Poverty				
< 185 % of FPL^e^	…….	…………….	1.00	Reference
185 %–349 % of FPL	……..	…………….	0.56	0.38, 0.87
> 350 %	-----	-------	1.05	0.65, 1.68
Parity				
Multipara	1.00	Reference	1.00	Reference
Primipara	0.57	0.43, 0.74	0.68	0.43, 1.07
Prenatal Smoking				
Current smokers	1.00	Reference	___	_____
Non smokers	2.32	1.29, 4.18	____	_____
Baby’s father support				
No	1.00	Reference	1.00	Reference
Yes	1.50	1.13, 1.99	1.77	1.04, 3.02
Maternal Obesity				
Non-obese	1.00	Reference	-----	------
Obese	0.67	0.52, 0.88	-----	------
Delivery Mode				
Vaginal	1.00	Referenc	1.00	Reference
Planned C-section	0.73	0.54, 0.99	0.84	0.51, 1.39
Emergency C-section	0.54	0.37, 0.79	0.37	0.17, 0.80
Return to work				
< 6 weeks	1.00	Reference	1.00	Reference
> 6 weeks	1.06	0.72, 1.56	1.52	0.57, 4.05
No plan to work	1.61	1.07, 2.41	4.26	1.65, 10.99

^a^Data source: Infant Feeding Practices Survey II, 2005–2007

^d^CI = confidence interval. ^b^EBF = Exclusive breastfeeding

^c^AOR= Adjusted Odds Ratio

^e^FPL= Federal Poverty Level: a measure of income level for determining financial eligibility for some welfare programs and benefits

In addition to ‘valued exclusive breastfeeding’ a number of factors were significantly associated with exclusively breastfeeding to at least 3 months. These include baby’s father support of breastfeeding (AOR 1.50; 95 % CI 1.13, 1.99), maternal education (AOR 1.55; 95 % CI 1.08, 2.21), not smoking vs smoking in the prenatal period (AOR 2.32; 95 % CI 1.29, 4.18) and having no plans to return to work (AOR 1.61; 95 % CI 1.07, 2.41). Maternal obesity (AOR 0.67; 95 % CI 0.52, 0.88) and cesarean delivery (AOR 0.54; 95 % CI 0.37, 0.79) were associated with reduced odds of exclusive breastfeeding to 3 months. Significant predictors of exclusive breastfeeding to 6 months were baby’s father support of exclusive breastfeeding (AOR 1.72; 95 % CI 1.01, 2.92), cesarean delivery (AOR 0.37; 95 % CI 0.17, 0.80) having no plans to return to work (AOR 4.26; 95 % CI 1.65, 10.99) and being middle income (185 to 345 % FPL) vs low income (< 185 % FPL) (AOR 0.56; 95 % CI 0.38, 0.87) (Table 3).

## Discussion

We found that a minority of mothers strongly value the benefits of exclusive breastfeeding, even among those who intend to exclusively breastfeed in the first few weeks postpartum. These mothers, nonetheless, had significantly higher odds of exclusively breastfeeding for at least 3 months and for 6 months compared to those who did not strongly value exclusive breastfeeding. These results suggest that valuing exclusive breastfeeding during pregnancy is a significant and independent determinant of exclusive breastfeeding duration.

Our study identified and measured attitudes toward exclusive breastfeeding among mothers who reported that they had an intention to exclusively breastfeed and found a strong association between attitude and behavioral intent. Whether the intent or the attitude came first remains to be determined. The results are consistent with our hypothesis that the extent to which a mother exclusively breastfeeds is strongly associated with the value she places on exclusive breastfeeding prenatally. The results of our analysis are also consistent with previous work that found maternal knowledge of the benefits of breastfeeding a determinant of breastfeeding initiation and duration [22, 36–39].

Approximately one-third of mothers in our analysis strongly valued exclusive breastfeeding, even though they have established intention to exclusively breastfeed; unfortunately, many of them still could not attain the recommended duration of breastfeeding. The CDC suggests that education about breastfeeding is the most effective single intervention to increase breastfeeding initiation and short-term duration, especially when it is delivered as part of a multicomponent intervention, tailored to personal needs of the mothers [40, 41]. It is a common perception that relative to its importance, breastfeeding is greatly undervalued; this perception may be due to the fact that not enough resources are devoted to promoting breastfeeding [3, 7]. Most women in the United States receive prenatal care, little of which currently consists of breastfeeding education. Many mothers may rely on friends and family members to provide advice which often may not be correct. The value that a mother places on the benefits of exclusive breastfeeding may be modifiable and can be a strong focus of breastfeeding intervention programs.

Our results also confirmed the importance of several factors previously associated with extended duration of exclusive breastfeeding [16, 32–34]. Support of baby’s father for exclusive breastfeeding, maternal education, not smoking and having no intention to return to work, were associated with higher odds of a baby being exclusively breastfed for longer durations. Obese mothers as well as mothers who had emergency cesarean section deliveries reported the least odds of exclusive breastfeeding for 3 or 6 months [33, 34]. Only few mothers who intended to exclusively breastfeed in the first few weeks postpartum and who reported strong value of exclusive breastfeeding could exclusively breastfeed for 3 months. These mothers may be challenged by environmental barriers to breastfeeding at home, workplace or hospitals. Such barriers are potentially modifiable factors, which together with targeted education to increase maternal knowledge of benefits of exclusive breastfeeding, may improve exclusive breastfeeding practices.

Our results have implications for practice. It would be useful to educate mothers about the benefits of exclusive breastfeeding during pregnancy. The most effective means of delivering this education may vary with different population groups. Repetitive, well structured, brief and illustrative prenatal education that extends into the postnatal period has been reported to be effective [42, 43]. Some intervention programs based on standard breastfeeding advice are associated with significant changes in knowledge, breastfeeding initiation and duration but not in exclusive breastfeeding rates [44, 45]. More effective results have been reported when maternal education is given as part of a multicomponent intervention which includes breastfeeding support from family, hospital and at work place that is tailored to the needs of the mothers in both the pre- and post-natal periods [38, 40, 43, 46].

From a policy perspective, it would be useful to consider requiring health care providers to deliver comprehensive lactation education during the prenatal and early postnatal period. Educational content should be comprehensive and offered in diverse settings including high schools, work sites and community centers. The current practice of health care providers delivering lactation education that varies from one facility to another with little or no emphasis on exclusive breastfeeding needs to be addressed. Breastfeeding education is so vital that it should be incorporated into any public health program that serves women and new families such as women’s health programs; teenage pregnancy programs; and Early Head Start programs. Inclusion of exclusive breastfeeding education in the curriculum for high school students may be promising [47]. The Affordable Care Act, which includes provisions to encourage breastfeeding education and its exclusivity, is a right step in the right direction. Environmental policies that enhance workplace and hospital breastfeeding supports are also important considerations.

Our study has limitations. The study population was drawn from a consumer opinion mail panel and thus not representative of mothers and infants in the United States. The mothers were predominantly white (84 % compared to 72 % nationally in 2010) [29], older, and more likely to be well educated and employed with fewer children. Participants were also less likely to smoke than mothers of infants born in 1998–2000 [27]. Although the IFPS II over sampled disadvantaged mothers (illiterate, non-English speaking, very low-income, very low education and without a stable home) compared to IFPS I, the results of our study may best describe practices of middle class American mothers rather than of disadvantaged American mothers [28]; thus, results cannot be generalized to all women in the United States. The reported exclusive breastfeeding rate may be higher than the national average as the sample consisted of women with established intension to breastfeed exclusively. Also, because of the small number of non-white women, we could not analyze results by race/ethnicity groups other than “non-white.” Maternal self-report of data could have been prone to bias and non-differential misclassification because of the tendency of women to be influenced by their social preferences and or social desirability; thus, women may have over-reported their value and duration of exclusive breastfeeding.

We used data from the IFPS II collected in 2005 to 2007, the most recent data available from this survey. There is no evidence of substantial changes between the time frame of the IFPS II and current breastfeeding practices in the United States. There was a slow increase in the breastfeeding behaviors from 2007 to 2011. However, the 2013 HealthStyles Survey showed that almost 40 % of the women surveyed were not convinced about the superiority of breastmilk over infant formula [48].

Our study also has several strengths. The prospective design of the IFPS II, the large sample size, as well as the extensive information on infant and maternal dietary practices make it useful for studying exclusive breastfeeding practices and testing hypotheses in a prospective manner [27]. Although the proportion of women who exclusively breastfed for up to 6 months is relatively small, the IFPS II is one of the largest data sources that include information about exclusive breastfeeding. The infant feeding practices recall period of 7 days minimized recall error. However, a potential consideration is that mothers received the neonatal questionnaire 3 weeks after delivery, which may introduce some degree of recall error of exclusive breastfeeding rates in the first 2 weeks of life. Finally, unlike most previous research, we studied women with very strong value for breastfeeding and controlled for many more confounding variables. These factors increase the validity of our results.

## Conclusion

Increasing exclusive breastfeeding rates and duration is an important public health challenge in the United States. Prenatal maternal education and environmental support that extends into the postnatal period can promote longer duration of exclusive breastfeeding [38]. More research is needed to determine the best way and time to deliver maternal education for maximal impact with special attention to the role of physicians, nurses and lactation experts [14, 15, 19, 37]. Investing in programs that focus on maternal education of exclusive breastfeeding benefits would help mothers attain their breastfeeding goals and/or the expert recommended exclusive breastfeeding durations.

## Abbreviations

AOR: adjusted odds ratio
CDC: centers for disease control and prevention
CI: confidence interval
FDA: food and drug administration
IFPS: infant feeding practices study

## References

[1] US Department of Health and Human Services : Maternal, infant and child health. Healthy People 20/20: Topics and objectives. 2010. Office of disease prevention and health promotion. Washington, DC. Available at https://www.healthypeople.gov/2020/topics-objectives/topic/maternal-infant-and-child-health/objectives. Accessed 04 January 2016.

[2] Eidelman AI (2012). Breastfeeding and the use of human milk: an analysis of the american academy of pediatrics 2012 breastfeeding policy statement. Breastfeed Med.

[3] Bai YK, Middlestadt SE, Joanne Peng CY, Fly AD (2009). Psychosocial factors underlying the mother’s decision to continue exclusive breastfeeding for 6 months: an elicitation study. J Hum Nutr Diet.

[4] CDC (2013). Progress in increasing breastfeeding and reducing racial/ethnic differences - united states, 2000-2008 births. Morbidity Mortal Week Report.

[5] McGuire S (2011). U.S. Dept. of health and human services. The surgeon General’s call to action to support breastfeeding. U.S. Dept. Of health and human services, office of the surgeon general. 2011.. Adv Nutr.

[6] Labbok M, Taylor E (2008). Achieving exclusive breastfeeding in the United States: findings and recommendations.

[7] Baker S, Claeson M, Schultink W. Inside the politics of breastfeeding promotion. Impatient Optimists, 2014. Available at http://www.impatientoptimists.org/Posts/2014/02/Inside-the-Politics-of-Breastfeeding-Promotion##. Accessed 14 March 2015.

[8] Center for Disease Control and Prevention: Breastfeeding among US children born 2001-2011. CDC National Immunization Survey. 2015. Available from: http://www.cdc.gov/breastfeeding/data/NIS_data/. Accessed 10 November 2015.

[9] World Health Organization: Breastfeeding Advocacy Initiative. For the Best Start in Life. 2015:1-9. WHO/NMH/NHD/15.1. Available at http://www.unicef.org/nutrition/files/Breastfeeding_Advocacy_Strategy-2015.pdf. Accessed 04 January 2016.

[10] DiGirolamo A, Thompson N, Martorell R, Fein S, Grummer-Strawn L (2005). Intention or experience? predictors of continued breastfeeding. Health Educ Behav.

[11] Odom EC, Li R, Scanlon KS, Perrine CG, Grummer-Strawn L (2013). Reasons for earlier than desired cessation of breastfeeding. Pediatrics.

[12] Kools EJ, Thijs C, Kester AD, de Vries H (2006). The motivational determinants of breast-feeding: predictors for the continuation of breast-feeding. Prev Med.

[13] Hamade H, Chaaya M, Saliba M, Chaaban R, Osman H (2013). Determinants of exclusive breastfeeding in an urban population of primiparas in Lebanon: a cross-sectional study. BMC Public Health.

[14] Racine EF, Frick K, Guthrie JF, Strobino D (2009). Individual net-benefit maximization: a model for understanding breastfeeding cessation among low-income women. Maternal Child Health J.

[15] Quarles A, Williams PD, Hoyle DA, Brimeyer M, Williams AR (1994). Mothers’ intention, age, education and the duration and management of breastfeeding. Matern Child Nurs J.

[16] Mueffelmann RE, Racine EF, Warren-Findlow J, Coffman MJ (2015). Perceived infant feeding preferences of significant family members and mother’s intention to exclusively breastfeed. J Hum Lact.

[17] Spencer BS, Grassley JS (2013). African American women and breastfeeding: an integrative literature review. Health Care Women Int.

[18] Motee A, Ramasawmy D, Pugo-Gunsam P, Jeewon R (2013). An assessment of the breastfeeding practices and infant feeding pattern among mothers in Mauritius. J Nutr Metab.

[19] Whalen B, Cramton R (2010). Overcoming barriers to breastfeeding continuation and exclusivity. Curr Opin Pediatr.

[20] Li R, Fein SB, Chen J, Grummer-Strawn LM (2008). Why mothers stop breastfeeding: mothers’ self-reported reasons for stopping during the first year. Pediatrics.

[21] Smith JP, Forrester R (2013). Who pays for the health benefits of exclusive breastfeeding? An analysis of maternal time costs. J Hum Lact.

[22] Hannon PR, Willis SK, Bishop-Townsend V, Martinez IM, Scrimshaw SC (2000). African-American and Latina adolescent mothers’ infant feeding decisions and breastfeeding practices: a qualitative study. J Adolesc Health.

[23] Mulready-Ward C, Hackett M (2014). Perception and attitudes: breastfeeding in public in New York City. J Hum Lact.

[24] Kronborg H, Vaeth M (2004). The influence of psychosocial factors on the duration of breastfeeding. Scandinavian J Public Health.

[25] Ward M, Sheridan A, Howell F, Hegarty I, O’Farrell A (2004). Infant feeding: factors affecting initiation, exclusivity and duration. Ir Med J.

[26] Seidel AK, Schetzina KE, Freeman SC, Coulter MM, Colgrove NJ (2013). Comparison of breast-feeding knowledge, attitudes, and beliefs before and after educational intervention for rural Appalachian high school students. South Med J.

[27] Fein SB, Labiner-Wolfe J, Shealy KR, Li R, Chen J, Grummer-Strawn LM (2008). Infant feeding practices study II: study methods. Pediatrics.

[28] FDA. Supporting Statement for OMB Review - Infant Feeding Practices Study II. Department of Health and Human Services. 2004. Available at www.fda.gov/OHRMS/DOCKETS/98fr/2004n-0166-ss00001.pdf. Accessed January 04, 2016

[29] Perrine CG, Scanlon KS, Li R, Odom E, Grummer-Strawn LM (2012). Baby-Friendly hospital practices and meeting exclusive breastfeeding intention. Pediatrics.

[30] Lopez AA, Higgins ST, Heil SH, Skelly JM, Lynch ME, Solomon LJ (2014). Relationships between postpartum cigarette smoking and breastfeeding duration. Drug Alcohol Depend.

[31] Bahadori B, Riediger ND, Farrell SM, Uitz E, Moghadasian MF. Hypothesis: Smoking decreases breast feeding duration by suppressing prolactin secretion. Med Hypotheses. 2013;81(4):582–6. 10.1016/j.mehy.2013.07.007 23948597

[32] Dewey KG, Nommsen-Rivers LA, Heinig MJ, Cohen RJ (2003). Risk factors for suboptimal infant breastfeeding behavior, delayed onset of lactation, and excess neonatal weight Loss. Pediatrics.

[33] Ahluwalia IB, Li R, Morrow B (2012). Breastfeeding practices: does method of delivery matter?. Matern Child Health J.

[34] Hauff LE, Leonard SA, Rasmussen KM (2014). Associations of maternal obesity and psychosocial factors with breastfeeding intention, initiation, and duration. Am J Clin Nutr.

[35] Amir LH, Donath S (2007). A systematic review of maternal obesity and breastfeeding intention, initiation and duration. BMC Pregnancy Childbirth.

[36] Murimi M, Dodge CM, Pope J, Erickson D (2010). Factors that influence breastfeeding decisions among special supplemental nutrition program for women, infants, and children participants from Central Louisiana. J Am Dietetics Assoc.

[37] Kornides M, Kitsantas P (2013). Evaluation of breastfeeding promotion, support, and knowledge of benefits on breastfeeding outcomes. J Child Health Care.

[38] Skouteris H, Nagle C, Fowler M, Kent B, Sahota P, Morris H (2014). Interventions designed to promote exclusive breastfeeding in high-income countries: a systematic review. Breastfeed Med.

[39] Seifu W, Assefa G, Egata G. Prevalence of exclusive breastfeeding and its predictors among infants aged 6 months in Jimma town, Southwest Ethiopia, 2013. J Pediatrics Neonatal Care. 2014;1(3):00017.

[40] CDC. Strategies to Prevent Obesity and other Chronic Diseases: The CDC Guide to Strategies to Support Breastfeeding Mothers and Babies. Atlanta: U.S. Department of Health and Human Services; 2013.

[41] Shealy KR, Li R, Benton Davis S, Grummer Strawn L. The CDC guide to breastfeeding interventions. Atlanta: United States Department of Health and Human Services, Centers for Disease Control and Prevention. 2005.

[42] Merewood A (2014). Prenatal education: timing it right. J Hum Lact.

[43] Chung M, Raman G, Trikalinos T, Lau J, Ip S (2008). Interventions in primary care to promote breastfeeding: an evidence review for the U.S. Preventive services task force. Ann Intern Med.

[44] Bonuck KA, Trombley M, Freeman K, McKee D (2005). Randomized, controlled trial of a prenatal and postnatal lactation consultant intervention on duration and intensity of breastfeeding up to 12 Months. Pediatrics.

[45] Khresheh R, Suhaimat A, Jalamdeh F, Barclay L (2011). The effect of a postnatal education and support program on breastfeeding among primiparous women: A randomized controlled trial. Int J Nurs Stud.

[46] Renfrew MJ, McCormick MF, Wade A, Quinn B, Dowswell T (2012). Support for healthy breastfeeding mothers with healthy term babies. Cochrane Database Syst Rev.

[47] Dyson L, Renfrew MJ, McFadden A, McCormick F, Herbert G, Thomas J (2010). Policy and public health recommendations to promote the initiation and duration of breast-feeding in developed country settings. Public Health Nutr.

[48] Center for Disease Control and Prevention. HealthStyles Survey : Public Beliefs and Attitudes About Breastfeeding. Division of Nutrition Physical Activity and Obesity. 2013. Available at http://www.cdc.gov/breastfeeding/data/healthstyles_survey/survey_2013.htm. Accessed 08 June 2015.

